# Probing impact on magnetic behavior of cobalt layer grown on thick MoS_2_ layer

**DOI:** 10.1038/s41598-024-54316-1

**Published:** 2024-03-01

**Authors:** Zainab Hussain, Shashikant P. Patole, Shoyebmohamad F. Shaikh, P. E. Lokhande, Habib M. Pathan

**Affiliations:** 1 Advanced Physics Laboratory, Department of Physics, Savitribai Phule Pune University, Pune, 411007 India; 2 Department of Physics, Khalifa University of Science and Technology, P.O. Box 127788, Abu Dhabi, United Arab Emirates; 3 Department of Chemistry, College of Science, King Saud University, P.O. Box 2455, 11451 Riyadh, Saudi Arabia; 4 Departamento de Mecánica, Facultad de Ingeniería, Universidad Tecnológica Metropolitana, Santiago, Chile

**Keywords:** Magnetic properties and materials, Spintronics, Surfaces, interfaces and thin films, Two-dimensional materials

## Abstract

Understanding the metal-semiconductor heterostructure interface is crucial for the development of spintronic devices. One of the prospective candidates and extensively studied semiconductors is molybdenum disulfide (MoS_2_). Herein, utilizing Kerr microscopy, we investigated the impact of thick MoS_2_ on the magnetic properties of the 10 nm Co layer. A comparative study on $\hbox {Co}/\hbox {MoS}_{2}$ and Co/Si shows that coercivity increased by 77% and the Kerr signal decreased by 26% compared to Co grown on Si substrate. In addition, the Co domain structure significantly changed when grown on MoS_2_. The plausible reason for the observed magnetic behavior can be that the Co interacts differently at the interface of MoS_2_ as compared to Si. Therefore, our studies investigate the interfacial effect on the magnetic properties of Co grown on thick MoS_2_ layer. Furthermore, our results will help in developing next-generation spintronic devices.

## Introduction

State-of-the-art spintronic devices rely substantially on recent advances in understanding the metal-semiconductor heterostructure interface. The last few decades have witnessed unprecedented advancement in using 2D transition metal dichalcogenides (TMDC) materials as semiconductors in various next-generation storage, sensors, and flexible electronics devices^1^. 2D TMDC materials have fascinating properties like direct band-gap at monolayers, flexibility, and tunable optical and electronic properties^1,2^. On the other hand, heterostructures composed of ferromagnetic and TMDC materials have great importance as they provide spin-orbit torque and consequently can be utilized in magnetic tunnel junction (MTJ) for charge-to-spin conversion^2^. Heavy materials, like Pt, Ta, etc., are typically used as spin torque due to their large spin-orbit coupling^3^. However, heavy metal/ferromagnetic junctions cannot be efficiently utilized for out-of-plane magnetic anisotropy magnetic tunnel junctions, and their charge-spin conversion efficiency is less^2,3^. Nevertheless, TMDC/FM heterostructure can be utilized as efficient charge-spin conversion for out-of-plane magnetic MTJ. Besides this, recent advancement shows that TMDC materials have great potential in low-powered spintronic devices because of the higher spin-orbit torque efficiency^3,4^.

The most widely studied 2D TMDC material is MoS_2_. Recently, various experimental evidence shows that MoS_2_ not only contributes in inducing the perpendicular anisotropy but also aids in inducing in-plane anisotropy and coercivity enhancement^5–8^. Even experimental and theoretical reports suggest that hybridization and low symmetry at the interface can lead to various exotic magnetic behaviors^9,10^. Currently, in literature, there have been reports on FM layer/monolayer MoS_2_ heterostructure^5,6,11,12^. Furthermore, in the past few years, scientists have extensively studied and explored monolayer and multilayer 2D TMDC/FM heterstuctures^11,13–16^. In a few such studies, one can find the investigation of Fe deposited on MoS_2_, which suggests that Fe does not react or bond with MoS_2_ and aggregates^11,17–19^. Nonetheless, a study on the Co grown on monolayer MoS_2_ shows that Co forms a covalent bond, and the spin magnetic moment also decreases^11^. This hybridization leads to the induced magnetic anisotropy in the Co layer^11^. Further, a theoretical report shows the hybridization of Co and S at the interface of $\hbox {Co}/\hbox {MoS}_{2}$ hetero-junctions^20^. Therefore, this suggests that one can tailor the magnetic behavior of the suitable FM layer, i.e., the Co layer, with the proximity of the MoS_2_ layer.

**Figure 1:**
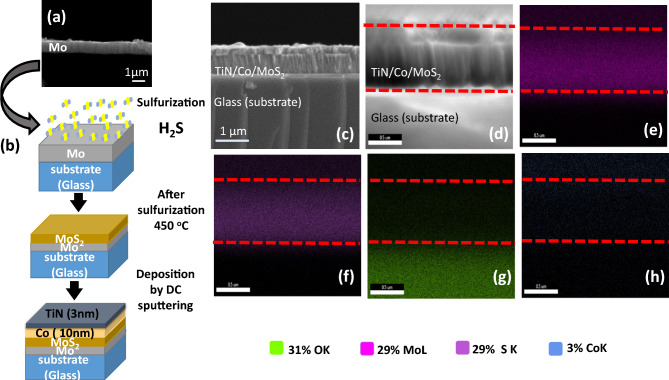
(**a**) Displays the SEM image of Mo thin film. (**b**) Illustrate the process of the sulfurization and deposition. (**c**) Displays the FE-SEM image of $\hbox {Co}/\hbox {MoS}_{2}$ (**d**–**h**), Display the EDS images.

Apart from this, the interfacial effect on magnetic properties of the FM layer grown on the thick MoS_2_ layer has received little attention, even though the scalability of the 2D TMDC layer is essential for application purposes. Contrarily, instead of utilizing techniques such as mechanical exfoliation or chemical vapor deposition to fabricate 2D TMDC layers, physical vapor deposition (sputtering) or direct sulfurization can produce homogeneous large-scale thick 2D TMDC layer^21,22^. It should also be noted that the wettability of the thick MoS_2_ increases with thickness as comparable to monolayer^23^. Hence, it is essential to investigate the interfacial effect of thick MoS_2_ on the magnetic properties of the FM layer. Consequently, it would be interesting to study the impact of the thick MoS_2_ interface on the Co layer. Therefore, our work provides an understanding of the impact on the magnetic behavior of the Co grown on the thick MoS_2_ layer prepared by direct sulfurization technique. Magneto-optical Kerr microscopy is employed in the present work to get valuable insights into magnetic behavior.

**Figure 2:**
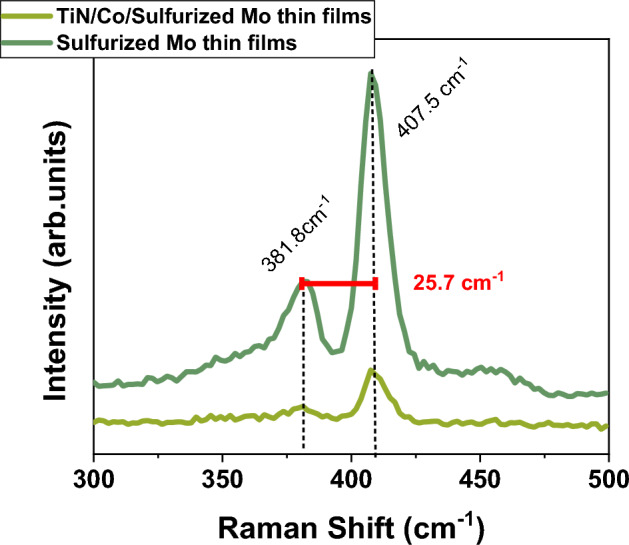
Displays the Raman spectra before and after the deposition of Co layer. One can clearly see the presence of two characteristic peaks confirming the formation of MoS_2_.

## Experimental

The MoS_2_ layer was prepared by sulfurization of commercially purchased Mo thin film deposited on glass substrate. The sulfurization was carried in tube type furnace by annealing at $450^{\circ }\hbox {C}$ temperature with 95% of argon and 5% of H_2_S gases for 1 hour. On other hand, the deposition of the Co layer on as-prepared MoS_2_ was carried out by using a DC magnetron sputtering (dcMS) system at the rate of 0.2Å per second at 50 watt. A capping layer of TiN ( $\approx$ 3nm) was deposited on the top of Co layer to avoid oxidation. Prior to the deposition, the as-prepared MoS_2_ sample was cleaned by RF biasing at 50 watt for 10 minutes for the removal of the contamination on the MoS_2_. Further, to compare the magnetic properties, Si substrate was mounted adjacent to MoS_2_. Thus, the deposition conditions were same for both the samples. The Raman spectroscopy was carried on the as-prepared sample by using cyan laser. Also scanning election microscopy (SEM) and field emission scanning electron microscopy along with energy dispersive spectroscopy (EDS) were performed on the samples. Moreover, atomic force microscopy (AFM) in tapping mode was performed to study the topography of the as-deposited samples . Imaging of the magnetic domains and the study of the magnetic anisotropy in the films was conducted by magneto-optical Kerr microscopy (M/s Evico Magnetics, Germany). Hysteresis loops were obtained by deriving the magnetization signal from the average domain image intensity.

**Figure 3:**
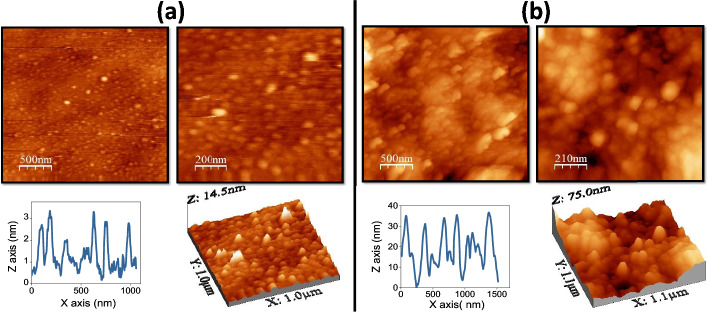
Displays the AFM images of the (**a**) TiN/Co/Si (**b**) TiN/$\hbox {Co}/\hbox {MoS}_{2}$ at different resolutions. Below frames, shows the line profiles and 3-D image of the TiN/Co/Si and TiN/$\hbox {Co}/\hbox {MoS}_{2}$ respectively.

**Figure 4:**
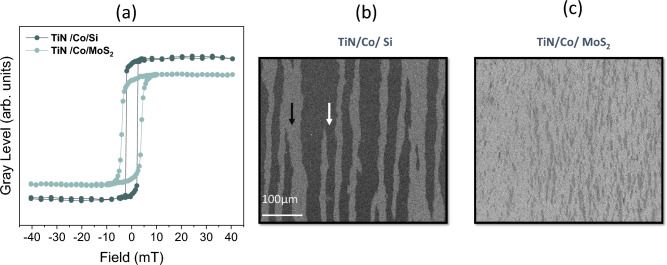
Displays the hysteresis loop for Co/Si and $\hbox {Co}/\hbox {MoS}_{2}$ and the corresponding domain structure captured at remanence state.

## Results and discussions

The MoS_2_ layer was prepared by sulfuring the sputtered Mo thin film of around 780± 39 $\mu$m thick ( confirmed by the SEM image in Fig. 1a). The sulfurization process is illustrated in Fig. 1b. It should be noted that in literature, large-scale MoS_2_ can be fabricated by various techniques such as sputtering, chemical vapor deposition, etc.^24^. However, sulfurization using sulfurization agent H_2_S gas is a simple and effective technique^24,25^. Consequently, we have selected H_2_S gas for the sulfurization of the MoS_2_ followed by deposition of Co layer of 10 nm and capped with TiN. To elucidate the structural uniformity at the interface and for chemical characterization, we have performed field emission scanning microscopy (FE-SEM) along with energy dispersive spectroscopy (EDS) on the $\hbox {Co}/\hbox {MoS}_{2}$ heterostructure. Figure 1c displays the FESEM micrograph $\hbox {Co}/\hbox {MoS}_{2}$ heterostructure indicating uniform and compact structure. Further, two contrast (structure) is visible, indicating two distinct layers. It probably indicates that the top Mo layers are sulfurized only. This observation is confirmed by grazing angle X-ray diffraction measurements (supplementary). The corresponding EDS mapping is displayed in Fig. 1d–h, confirming the deposition of different layers like Co and MoS_2_. Although from the EDS contrast image, it is difficult to distinguish the distribution of Co and S precisely since the EDS resolution is limited by 1 micron. However, one can identify the presence of elements like Mo, Co, O, and S. The formation of MoS_2_ is confirmed by the Raman spectra in Fig. 2. One can clearly see the two characteristic peaks corresponding to in-plane ($\hbox {E}^{1}_{\textrm{2g}}$) and out-of-plane ($\hbox {A}_{\textrm{1g}}$) vibrational modes at 381.8 cm^-1^ and $407.5\,\hbox {cm}^{-1}$ respectively. The separation between two peaks is $\approx$ $25\,\hbox {cm}^{-1}$ indicating the bulk nature of MoS_2_ layer^1^. Besides this, the AFM is performed to study the morphology and surface of the as-deposited samples. Analyzing the topography of both samples by AFM can be helpful in providing information regarding the interface. Figure 3 shows the AFM images taken at different resolutions for TiN/Co/Si and TiN/Co/Si, respectively. The frames below show the 3-D image and the line profile for both samples. It is observed that the root mean square (RMS) roughness for both the samples are different, i.e., 0.64 nm for TiN/Co/Si and 15.1 nm for TiN/$\hbox {Co}/\hbox {MoS}_{2}$. It indicates that the roughness significantly increased when Co has grown MoS_2_ underlayer. Moreover, the grain size of the surface calculated by the line profile is 45 ± 8 nm for TiN/Co/Si, while 90 ± 11nm for TiN/$\hbox {Co}/\hbox {MoS}_{2}$. Thus, the AFM study indicated that the Co grows and interacts differently on the interface of the MoS_2_ as compared to the Si; this leads to modification in the morphology in $\hbox {Co}/\hbox {MoS}_{2}$.

**Figure 5:**
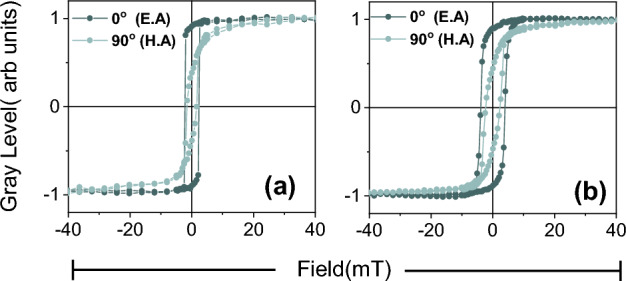
Displays the magnetic hysteresis loops measured along easy axis and hard axis which is designated as $0^o$ and $90^o$ for (**a**) TiN/Co/Si and (**b**)TiN/$\hbox {Co}/\hbox {MoS}_{2}$ respectively.

In order to investigate the magnetic behavior of as-deposited $\hbox {Co}/\hbox {MoS}_{2}$ heterostructure, we have performed Kerr microscopy. For comparison of magnetic properties, we have compared with the Co layer grown on the silicon substrate. The magnetic hysteresis loops measured along the magnetic domain structure for both the samples Co/Si and $\hbox {Co}/\hbox {MoS}_{2}$ is displayed in Fig. 4. One can clearly observe the increase in coercivity by 77% and a decrease in Kerr signal by 26% as compared to the Co/Si sample. The reduction in the Kerr signal signifies that the contribution from the magnetic moment is decreased^18,26^. It could be due to roughness or inhomogeneous microstructure at the interface. Further, increasing the coercivity indicates an increase in the pinning center due to inhomogeneity or roughness at the interface. To get deeper insights into the magnetic behavior, the magnetic domain structure was captured along the remanence state by applying a decreasing A.C. field along the easy axis of magnetization. From Fig. 4, a noteworthy difference in domain structure can be observed for both samples. The fine domains are observed in $\hbox {Co}/\hbox {MoS}_{2}$; contrarily, large domains are observed for Co/Si. The observation of fine magnetic domain for $\hbox {Co}/\hbox {MoS}_{2}$ can be attributed to the fact that the magnetic domains often split into smaller domains when the pinning centers are increased in order to reduce the magneto-static energy^27^. Moreover, domain structure corroborates the observation of increased coercivity in $\hbox {Co}/\hbox {MoS}_{2}$ compared with Co/Si.

**Table 1:** Parameters obtained from hysteresis loops ( in Fig. 5) is shown below in table.

Sample	E.A $H_C$ (mT)	H.A $H_C$ (mT)	$M_r/M_S$
TiN/Co/Si	2.2	1.5	0.38
TiN/$\hbox {Co}/\hbox {MoS}_{2}$	3.9	2.2	0.45

In an effort to study the in-plane magnetic anisotropy in both the samples. The hysteresis loops were measured along the in-plane azimuthal angles. Figure 5 displays the hysteresis loop measured along an easy axis (E.A.) and hard axis (H.A.), which is designated as $0^o$ and $90^o$. Apparently, both the samples, i.e., Co/Si and $\hbox {Co}/\hbox {MoS}_{2}$, possess magnetic anisotropy. Usually, the polycrystalline Co thin film possesses in-plane magnetic anisotropy due to various causes^28^. From Fig. 5, it is clear that MoS_2_ does not contribute much to the magnetic anisotropy. For comparison, the coercivity values ($H_C$ ) and squareness ($M_r$/$M_s$) for $0^o$ and $90^o$ shown in Table 1. One clearly observed that the $H_C$ is more for the $0^o$ and $90^o$ in $\hbox {Co}/\hbox {MoS}_{2}$ as compared to Co/Si.

## Conclusions

The impact on the magnetic properties of Co grown on thick MoS_2_ under layer is investigated with magneto-optical Kerr microscopy. The results are compared with the conventional Co thin films grown on Si substrate. Results show that $\hbox {Co}/\hbox {MoS}_{2}$, coercivity increased by 77%, and the Kerr signal decreased by 26% compared to Co grown on Si substrate. In addition, the Co domain structure significantly changed when grown on MoS_2_. The plausible reason for the observed magnetic behavior can be that the Co interacts differently at the interface of MoS_2_. This leads to a significant change in the morphology and magnetic behavior. Further, this aspect needs to be explored in more detail. Experiments like X-ray photoelectron spectroscopy can be utilized to study the chemical interaction and hybridization at the interface in such heterostructures.

### Supplementary Information

Supplementary Information.

## Data Availability

The datasets used and/or analyzed during the current investigation are accessible from the corresponding author on reasonable request.
