# Supplementary material for: Probing impact on magnetic behavior of cobalt layer grown on thick MoS2 layer
Source: Sci Rep. 2024 Mar 1;14:5064. doi: 10.1038/s41598-024-54316-1 (PMC10904856; doi:10.1038/s41598-024-54316-1)
Supplement: Supplementary file 1 — Supplementary Information. [file 41598_2024_54316_MOESM1_ESM.docx]

**Probing impact on magnetic behavior of cobalt layer grown on thick MoS_2_ layer**

***Zainab Hussain^1^, Shashikant P. Patole^2^, Shoyebmohamad F. Shaikh ^3^, P.E Lokhande^4^ and Habib M. Pathan^1^***

*^1^Advanced Physics Laboratory; Department of Physics; Savitribai Phule Pune University; Pune-411007, India.*

*^2^Department of Physics, Khalifa University of Science and Technology, P.O. Box 127788, Abu Dhabi, United Arab Emirates*

*^3^Department of Chemistry; College of Science, King Saud University; P.O. Box 2455; Riyadh 11451; Saudi Arabia*

*^4^Departamento de Mecnica, Facultad de Ingeniera, Universidad Tecnolgica Metropolitana, Santiago, Chile*


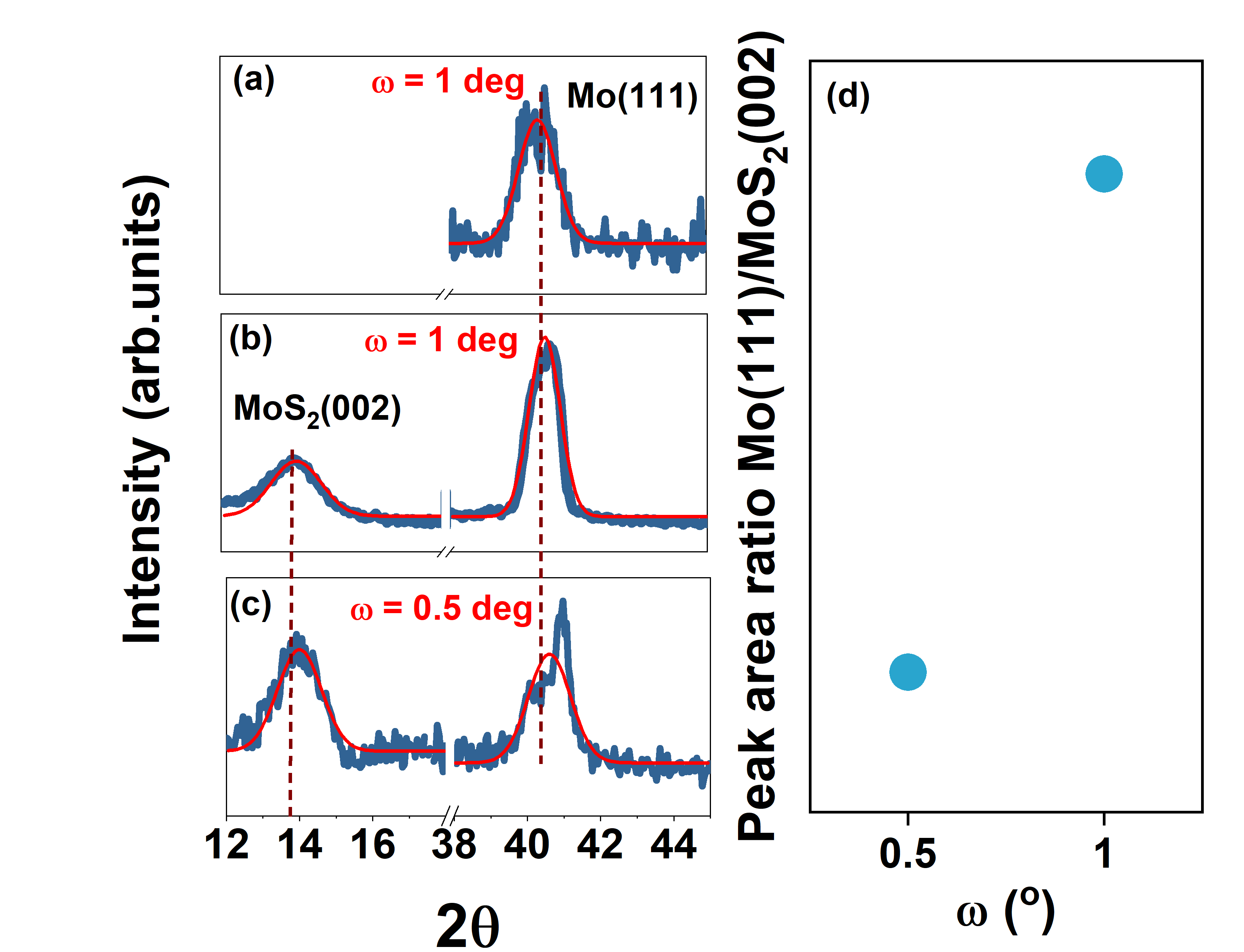
To corroborate FESEM data that the top layers of Mo thin film are converted into MoS_2_. We have performed the grazing angle X-ray diffraction (GIXRD). GIXRD measurement is an effective technique for depth phase profiling. X-ray penetration depth can be tuned by changing the grazing angle of incidence, i.e., the more the angle of incidence, the more the X-ray will penetrate, and vice versa. We have performed the GIXRD measurement on both the Mo thin film before (after) sulfurization at an angle of incidence (ω) = 0.5 ^o^ and 1^o^. Figure-1S (a) shows the before sulfurization for ω= 1^o^, and figure-1S (b-c) shows the GIXRD pattern after ω= 0.5^o^ and 1^o^. One observes the peak at 14.05^o^ corresponds to the MoS_2_ for the reflection plane (002), respectively. Further, we would observe the peak at 40.5^o^, corresponding to Mo for the reflection of the plane (111). One can notice that the peak intensity for the (111) plane of Mo increases with the angle of incidence, indicating that the bottom layer is more enriched with Mo. This can be quantified in Figure—1S (d), by estimating the peak area ratio of Mo (111)/MoS_2_ (002), which clearly indicates that the ratio of Mo (111)/MoS_2_ (002) increases with increasing the angle of incidence.

Figure-1S (a-c), Shows the GIXRD pattern obtained at ω= 0.5^o^ and 1^o^ for before (after) sulfurization of Mo thin film. (c), shows the peak area ratio of Mo(111)/MoS_2_(002)
